# Myocardial strain analysis of the right ventricle: comparison of different cardiovascular magnetic resonance and echocardiographic techniques

**DOI:** 10.1186/s12968-020-00647-7

**Published:** 2020-07-23

**Authors:** Jennifer Erley, Radu Tanacli, Davide Genovese, Natalie Tapaskar, Nina Rashedi, Paulius Bucius, Keigo Kawaji, Ilya Karagodin, Roberto M. Lang, Sebastian Kelle, Victor Mor-Avi, Amit R. Patel

**Affiliations:** 1 Department of Internal Medicine / Cardiology, German Heart Center, Berlin, Germany; 2 Department of Cardiac, Thoracic, Vascular Sciences and Public Health, University of Padua, Padua, Italy; 3 Department of Medicine, University of Chicago Medical Center, 5758 S. Maryland Avenue, MC9067, Chicago, IL 60637 USA; 4 Department of Biomedical Engineering, Illinois Institute of Technology, Chicago, IL USA; 5 Charité Campus Virchow Klinikum, Department of Internal Medicine/Cardiology, Berlin, Germany; 6 DZHK (German Center for Cardiovascular Research), Partner Site Berlin, Berlin, Germany

**Keywords:** Cardiovascular magnetic resonance, Speckle-tracking echocardiography, Right-ventricular function

## Abstract

**Background:**

Right ventricular (RV) strain is a useful predictor of prognosis in various cardiovascular diseases, including those traditionally believed to impact only the left ventricle. We aimed to determine inter-modality and inter-technique agreement in RV longitudinal strain (LS) measurements between currently available cardiovascular magnetic resonance (CMR) and echocardiographic techniques, as well as their reproducibility and the impact of layer-specific strain measurements.

**Methods:**

RV-LS was determined in 62 patients using 2D speckle tracking echocardiography (STE, Epsilon) and two CMR techniques: feature tracking (FT) and strain-encoding (SENC), and in 17 healthy subjects using FT and SENC only. Measurements included global and free-wall LS (GLS, FWLS). Inter-technique agreement was assessed using linear regression and Bland-Altman analysis. Reproducibility was quantified using intraclass correlation (ICC) and coefficients of variation (CoV).

**Results:**

We found similar moderate agreement between both CMR techniques and STE in patients: r = 0.57–0.63 for SENC; r = 0.50–0.62 for FT. The correlation between SENC and STE was better for GLS (r = 0.63) than for FWLS (r = 0.57). Conversely, the correlation between FT and STE was higher for FWLS (r = 0.60–0.62) than GLS (r = 0.50–0.54). FT-midmyocardial strain correlated better with SENC and STE than FT-subendocardial strain. The agreement between SENC and FT was fair (r = 0.36–0.41, bias: − 6.4 to − 10.4%) in the entire study group. All techniques except FT showed excellent reproducibility (ICC: 0.62–0.96, CoV: 0.04–0.30).

**Conclusions:**

We found only moderate inter-modality agreement with STE in RV-LS for both FT and SENC and poor agreement when comparing between the CMR techniques. Different modalities and techniques should not be used interchangeably to determine and monitor RV strain.

## Introduction

Myocardial strain is a useful diagnostic measurement to assess ventricular function. Left ventricular (LV) strain was found to have incremental prognostic value over routinely assessed parameters, such as ejection fraction (EF), in the context of the detection of inducible ischemia [1] and of heart failure with preserved ejection fraction [2], as well as the evaluation of chemotherapy-induced cardiotoxicity [3], among other indications. Recent studies demonstrated the value of right ventricular (RV) strain in diseases such as pulmonary hypertension [4] and pulmonary embolism [5], which primarily impact the RV, and also in heart failure [6–8] and congenital heart disease [9], RV strain was found to have independent additional prognostic value when compared to LV strain alone [6]. RV strain can be determined using cardiovascular magnetic resonance (CMR) and speckle tracking echocardiography (STE) [10, 11].

STE based RV strain can be measured globally or exclusively in the free wall by excluding the interventricular septum [12]. CMR is currently the gold standard for the determination of RV function and volume [13–15]. Feature tracking (FT) is one of the currently available CMR techniques to measure strain. Similar to STE, FT uses cine images to track points in the myocardium over the entirety of the cardiac cycle [16] by a dedicated algorithm, in order to measure myocardial strain within the imaging plane. Strain-encoding (SENC), a different CMR technique, uses through-plane tags to measure strain in the direction perpendicular to the imaging plane [17]. Hence, longitudinal strain (LS) is analyzed from long-axis images by FT and from the short-axis images by SENC.

Although there are several studies assessing RV strain, few have compared different imaging modalities and have produced conflicting results [4, 15, 18–22]. Furthermore, the impact of layer-specific strain on the inter-technique agreement has not yet been investigated. Moreover, there is no consensus whether RV strain analysis should include the septum to measure global longitudinal strain (GLS) or exclude the septum, resulting in free-wall longitudinal strain (FWLS). Finally, the reproducibility of the different approaches has not been systematically analyzed to allow for direct comparisons. Therefore, we aimed to: 1) investigate inter-modality agreement between FT- and SENC-derived RV LS and STE GLS and FWLS, 2) determine inter-technique agreement between FT and SENC in patients and in normal subjects; 3) study the reproducibility of these three techniques, and 4) analyze the impact of subendocardial versus midmyocardial FT strain measurements on the inter-technique agreement.

## Methods

### Study population

We retrospectively identified and analyzed 62 consecutive patients who underwent CMR imaging and transthoracic 2D echocardiography and who had the necessary images to assess RV strain using STE, FT, and SENC. The different imaging modalities were used within a median time of 3.5 days (interquartile range: 1–18 days). Patients were excluded if they were under the age of 18 or received any cardiac intervention in between the examinations. Additionally, the FT and SENC RV strain measurements were compared to those of 17 healthy subjects who only underwent CMR, including SENC. The Institutional Review Board approved this study with a waiver of consent.

### 2D speckle tracking echocardiography

**Fig. 1:**
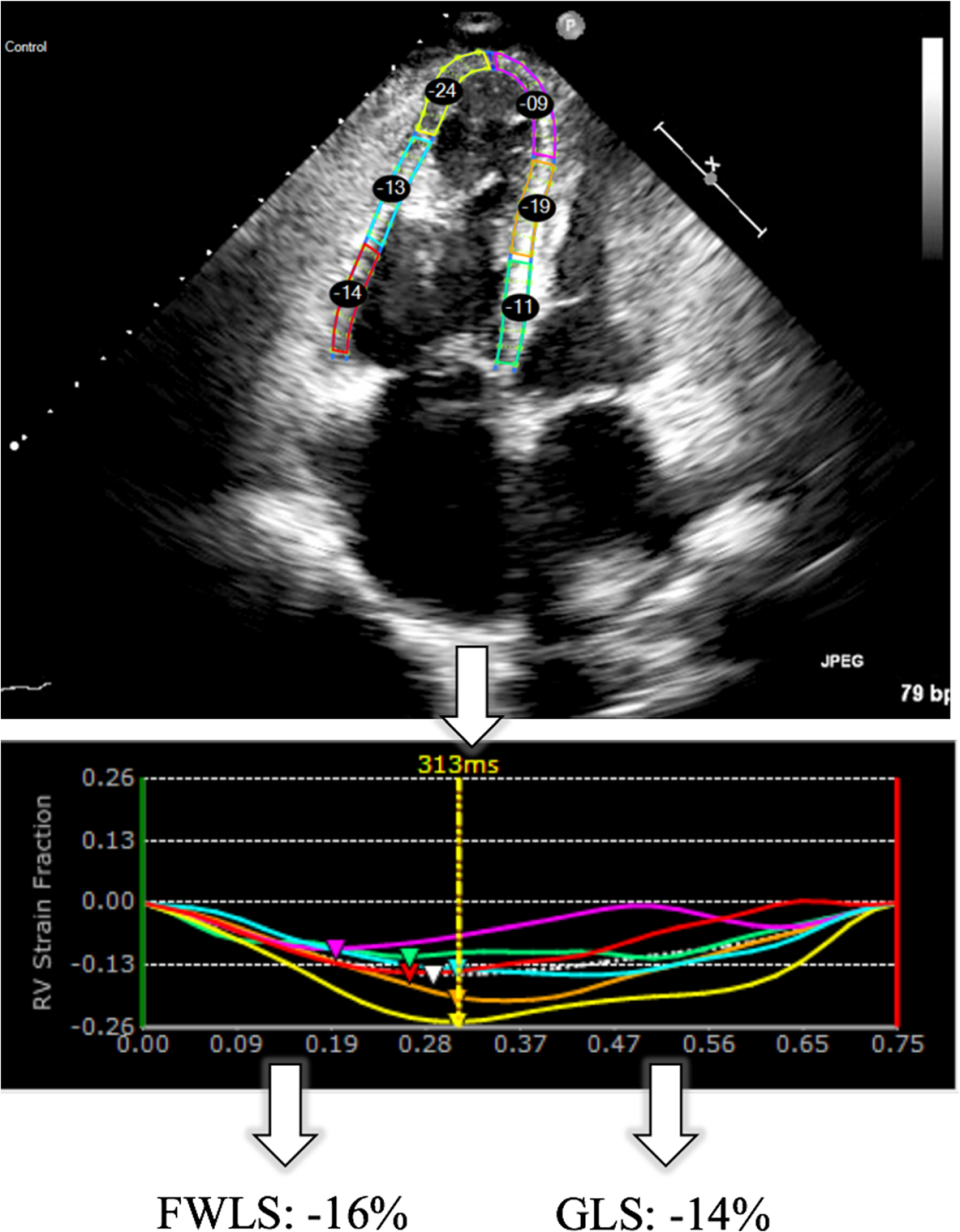
Representative speckle tracking echocardiography (STE)-image, strain curves and end-systolic strain values of a patient with pulmonary artery hypertension and right-sided heart failure (LVEF:56%, RVEF:28%). LVEF, left ventricular ejection fraction; RVEF, right ventricular ejection fraction. GLS = global longitudinal strain, FWLS = free wall longitudinal strain

Echocardiographic imaging was performed using an iE33 system with X5–1 probe (Philips Healthcare, Andover, Massachusetts, USA). RV-focused four-chamber (4Ch) views were acquired at a heart rate of 74 (±18) bpm, after optimizing the sector size, gain, depth, compress and time-gain compensation. Frame rate was maximized (76 ± 24 fps) by decreasing the depth and sector width. The images were stored digitally and measured offline according to the European Association of Cardiovascular Imaging/American Society of Echocardiography [23] by an experienced reader (DG), blinded to clinical data and all CMR strain measurements, using vendor independent speckle-tracking software (Echo Insight, Epsilon Imaging, Ann Arbor, Michigan, USA). End-diastole (ED) was identified as the frame coinciding with the peak of the QRS complex, whereas end-systole (ES) was identified as the frame with the smallest RV cavity. The region of interest was manually traced at ED along the endocardial border from the tricuspid valve annulus to the RV apex and back to the annulus. The software then automatically tracked the endocardial contours throughout the cardiac cycle. Manual adjustments were made to the contours as needed to optimize tracking. RV-GLS was calculated throughout the cardiac cycle, resulting in a time-strain curve (Fig. 1). Similarly, FWLS was determined by the software after excluding the septal strain values.

### CMR imaging

CMR images of the patients were acquired using a 1.5 T CMR scanner with a 5-channel surface coil (Achieva, Philips Healthcare, Best, the Netherlands). Retrospectively gated cine images were acquired using a balanced steady-state free precession (bSSFP) cine pulse sequence in the 2-,3-,4Ch and short-axis views. Scanning parameters were: TR = 2.9 ms, TE = 1.5 ms, flip angle 60°, temporal resolution 30-40 ms. SENC-images were acquired in three short-axis views (basal, mid-ventricular and apical) to determine RV-LS. Scanning parameters were: TR = 13 ms; TE = 0.7 ms; FA = 30°; field-of-view = 256x256mm^2^; slice thickness = 10 mm; 24 ms SENC magnetization preparation prior to continuous acquisition of 40 ms (3 spiral interleaves) per temporal frame over 1 R-R cycle.

### Feature tracking

**Fig. 2:**
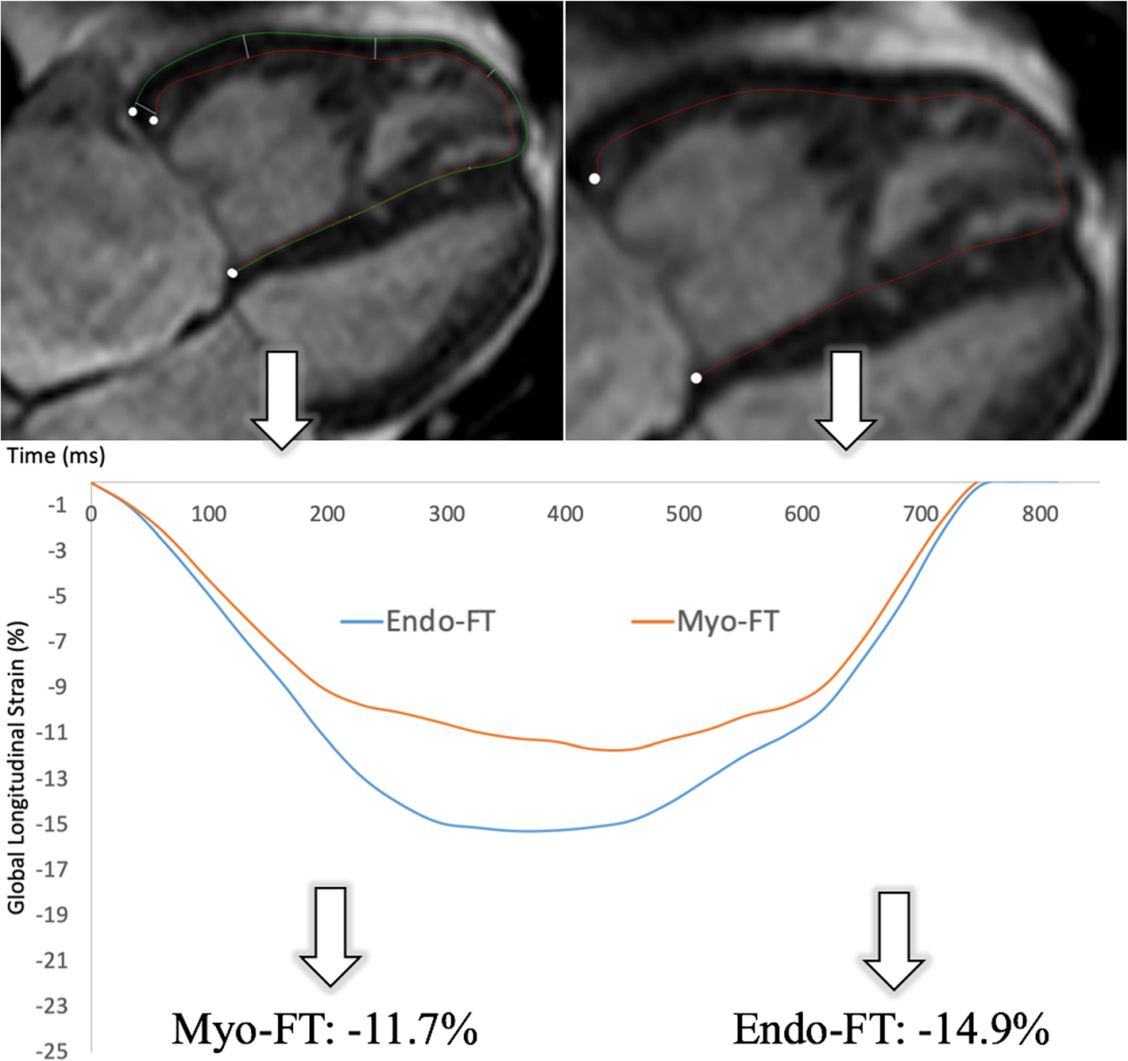
Representative feature tracking (FT)-images, demonstrating the two contouring approaches to determine subendocardial (Endo-FT) and midmyocardial (Myo-FT) strain, as well as the corresponding strain curves and end-systolic strain values for the same patient with pulmonary artery hypertension and right-sided heart failure

FT was performed offline by a different experienced observer (RT), blinded to clinical data and all other strain measurements, using vendor-independent software (*QStrain (Research Edition), Medis, Leiden, Netherlands*). RV LS was determined from the bSSFP long-axis 4-Ch view, excluding the septum. ED and ES were detected and the endocardial and epicardial contours were drawn from the tricuspid valve anulus to the apex of the RV and back to the opposite annulus and segmented respectively. Measurements were performed separately to determine subendocardial strain, only including the endocardial contour, and midmyocardial strain including both the epi- and endocardial contour **(**Fig. 2**)**.

### Strain-encoding

**Fig. 3:**
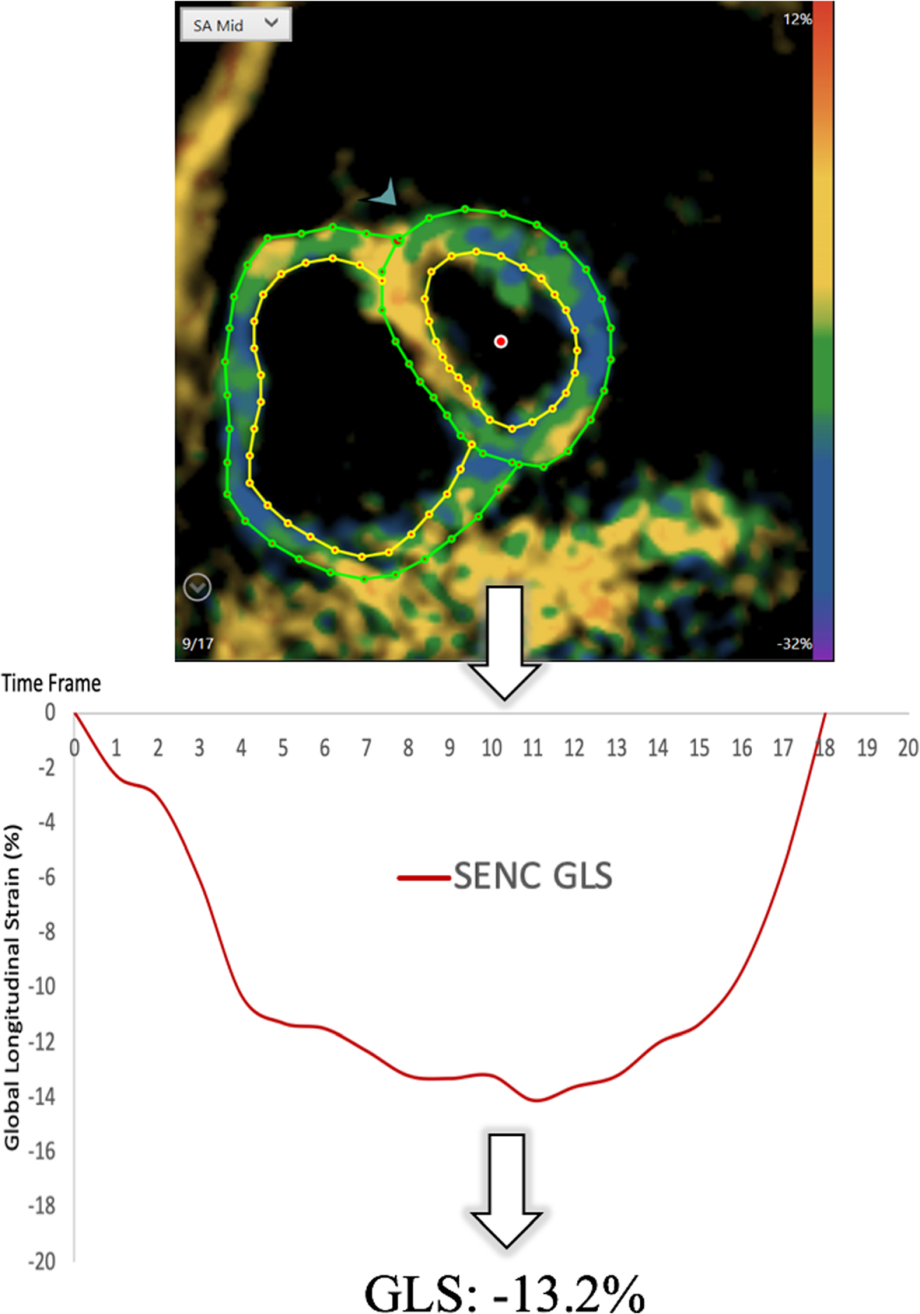
Representative strain encoding (SENC)-image, strain curve and end-systolic strain value of the same patient with pulmonary artery hypertension and right-sided heart failure. The color-coded images represent the myocardial contraction. GLS, global longitudinal strain

SENC images were analyzed by a third observer (JE), blinded to all previous strain analyses, using vendor-independent software (Myostrain 5.0, Myocardial Solutions, Morrisville, North Carolina, USA). The basal and mid-ventricular short-axis views were both used for strain analysis. The apical view could not be used as there is often not a significant amount of RV myocardium present at that level. Epi- and endocardial contours of the RV were drawn manually at ES after drawing the LV contours, starting at the contour of one RV insertion point into the septum and stopping at the opposite insertion point. The software automatically calculated LS at ES **(**Fig. 3**)**, excluding the septum.

### Reproducibility analysis

Strain analysis was repeated for all three modalities and techniques in a subset of 10 randomly selected patients by the same observer more than 2 weeks after the first analysis and by a second observer, different for each modality (STE by N.R., FT by P.B., SENC by N.T.), who was blinded to all previous strain analyses.

### Statistical analysis

Values were assessed for normality using the Shapiro-Wilk test. Normally distributed data are expressed as mean ± SD, non-normally distributed data using median and interquartile range (IQR). Correlation between STE, FT and SENC was determined using Pearson correlation coefficient (normally distributed) or Spearman-Rank (non-normally distributed). Inter-technique and inter-modality agreement were assessed using Bland-Altman analysis of biases and limits of agreement. The distribution of strain measurements between STE, FT and SENC in patients and between FT and SENC in the reference group of volunteers were determined using Friedmann’s test and Dunn-Bonferroni post hoc test. The effect strength of the differences in strain measurements was calculated using Pearson’s correlation coefficients (r), and interpreted as follows: 0.1 ≤ r < 0.3 = weak; 0.3 ≤ r < 0.5 = moderate; r > 0.5 = strong [24]. Intra- and inter-observer variability was expressed in terms of intraclass-correlations (ICC) and coefficients of variation (CoV). A *p*-value of ≤0.05 was considered significant in two-tailed tests. Statistical analyses were conducted using SPSS (Version 25.0, Statistical Package for the Social Sciences, International Business Machines, Inc., Armonk, New York, USA).

## Results

Four of the 62 patients had to be excluded due to an uploading error on the STE-platform and one patient had to be excluded due to FT artifacts, resulting in a total analysis of 57 patients. Table 1 shows the demographic characteristics, diseases and median strain values of the patients and the 17 healthy subjects.

**Table 1:** Demographic characteristics, diseases and median right ventricular strain values of the patients and volunteers

	Patients (*n* = 57)	Healthy subjects (*n* = 17)
Age (years)	56 (±19)	24 (±5)
Female, n (%)	33 (57.9%)	9 (53%)
LV-dysfunction (LVEF< 50%, e.g. HFrEF, CAD, arrhythmia), n (%)	17 (29.8%)	/
Preserved LVEF (LVEF> 50%, e.g. HFpEF, CAD, arrhythmia), n (%)	13 (22.8%)	/
Pulmonary hypertension, RV-dysfunction, n (%)	12 (21%)	/
Congenital heart disease, n (%)	8 (14%)	/
Systemic rheumatic/ inflammatory disease, n (%)	5 (8.8%)	/
Symptoms but no diagnosis on cardiac imaging, n (%)	2 (3.5%)	/
Median (IQR) BSA (m^2^)	1.9 (1.7–2.0)	1.8 (1.7–1.9)
LVEF (from CMR) (%)	51.2 (16.1)	58.6 (5.1)
RVEF (from CMR) (%)	51.8 (14.5)	53.4 (5.8)
LVEF < 50%	24 (42.1%)	0
RVEF< 50%	18 (31.6%)	2 (11.8%)
Median (IQR) GLS for STE	−16.0 (−20.0 to −14.0)	/
Median (IQR) FWLS for STE	−21.0 (−26.0 to −17.0)	/
Median (IQR) LS for Endo-FT	−25.2 (−32.5 to −21.6)	−27.6 (− 31.7 to −23.2)
Median (IQR) LS for Myo-FT	−26.3 (− 30.6 to − 20.6)	−25.6 (− 29.3 to − 22.4)
Median (IQR) LS for SENC	−18.0 (− 20.0 to − 15.8)	−18.6 (− 21.0 to − 17.7)

*Abbreviations: BSA* Body surface area, *LVEF* Left ventricular ejection fraction, *RVEF* Right ventricular ejection fraction, *IQR* Interquartile range, *GLS* Global longitudinal strain, *FWLS* Free wall longitudinal strain, *STE* Speckle tracking echocardiography, *SENC* Strain-encoding, *FT* Feature tracking, *Endo-FT* Subendocardial strain determined using FT, *Myo-FT* Midmyocardial strain determined using FT, *HFrEF* Heart failure and reduced ejection fraction, *HFpEF* Heart failure and preserved ejection fraction

### Inter-modality agreement

Table 2 displays the results of the Bland-Altman analysis and the correlation coefficients between the modalities and techniques. The agreement between either CMR technique and STE was moderate and very similar. The best correlation was found between SENC and STE-GLS, reflected by a very low bias. The correlation between Myo-FT and STE-FWLS was similarly high, but the bias between these techniques was larger. SENC agreed better with STE-GLS, whereas FT agreed better with STE-FWLS. Myo-FT correlated better with STE than Endo-FT and also resulted in a smaller bias and narrower limits of agreement.

**Table 2:** Inter-modality and inter-technique agreement for right ventricular strain measurements, shown by Bland-Altman analyses and correlation coefficients

*Patients (n = 57)*	r	p	Bias (%)	LOA (%)	p
SENC vs. STE					
SENC vs. STE-FWLS	0.57	< 0.001	−3.6	−12.2 to 5.0	< 0.001
SENC vs. STE-GLS	0.63	< 0.001	0.7	−5.3 to 6.8	0.094
FT vs. STE					
Endo-FT vs. STE -FWLS	0.60	< 0.001	6.8	−9.8 to 23.5	< 0.001
Myo-FT vs. STE-FWLS	0.62	< 0.001	5.5	−9.0 to 20.0	< 0.001
Endo-FT vs. STE-GLS	0.50	< 0.001	11.1	−6.6 to 28.9	< 0.001
Myo-FT vs. STE-GLS	0.54	< 0.001	9.8	−5.3 to 24.9	< 0.001
SENC vs. FT					
SENC vs. Endo-FT	0.39	0.003	−10.4	−28.8 to 8.0	< 0.001
SENC vs. Myo-FT	0.41	0.002	−9.1	−24.7 to 6.6	< 0.001
*Volunteers (n = 17)*					
SENC vs. FT					
SENC vs. Endo-FT	0.39	0.129	−8.3	−16.9 to 0.28	< 0.001
SENC vs. Myo-FT	0.36	0.162	−6.4	−14.1 to 1.30	< 0.001

*Abbreviations: GLS* Global longitudinal strain, *FWLS* Free wall longitudinal strain, *STE* Speckle tracking echocardiography, *SENC* strain-encoding, *FT* Feature tracking, *Endo-FT* Subendocardial strain determined using FT, *Myo-FT* Midmyocardial strain determined using FT, *LOA* Limits of agreement

### Inter-technique agreement

Inter-technique agreement between SENC and FT was poor and very similar in patients and in healthy subjects, reflected by a low correlation and a substantial bias with wide limits of agreement.

### Distribution of strain measurements in patients and in healthy subjects

**Fig. 4:**
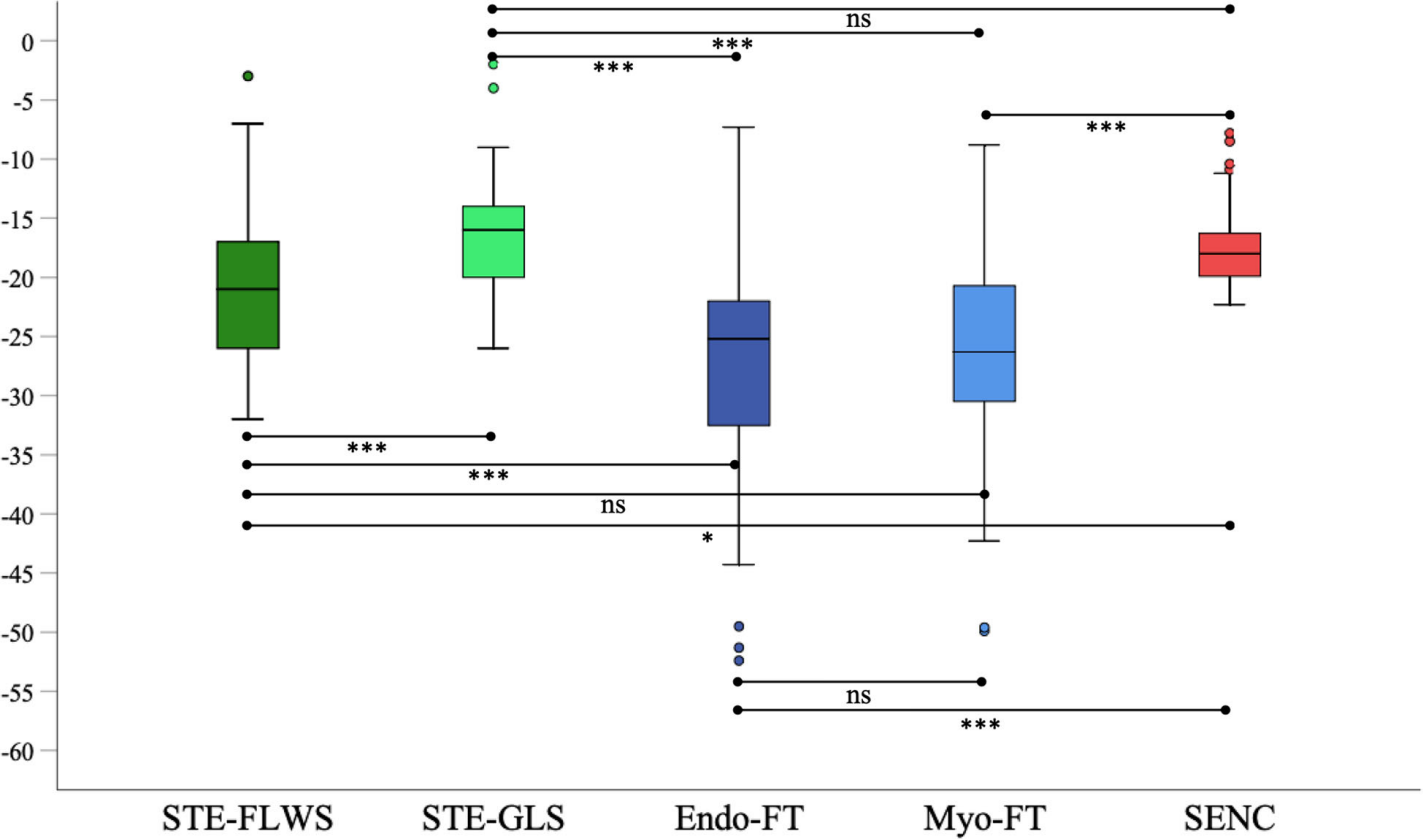
Box-plot diagrams illustrating the distribution of strain values, determined using STE, FT and SENC in 57 patients. Ns, not significant; * = *p* < 0.05; *** = *p* < 0.001

**Fig. 5:**
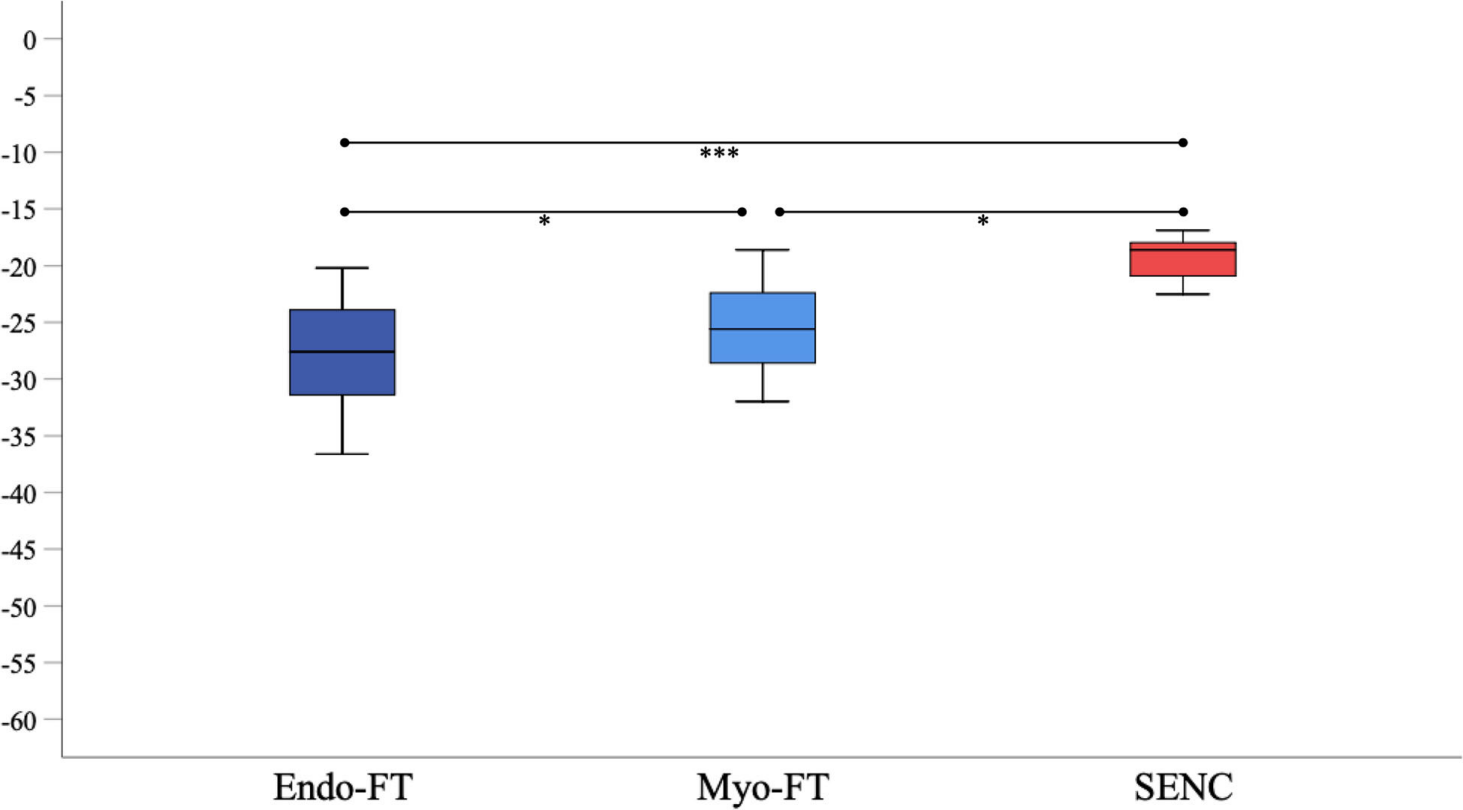
Box-plot diagrams illustrating the distribution of strain values, determined using FT and SENC in the reference group of 17 healthy subjects. * = p < 0.05, *** = p < 0.001

Figures 4 and 5 show box-plots depicting the distribution of strain measurements in patients and healthy subjects, as well as the significance level of the Dunn-Bonferroni post-hoc test. Table 3 displays the results of the Friedmann analysis, and the significance level determined in the Dunn-Bonferoni test for every pairwise comparison, as well as the effect size of the different comparisons. Both in patients and in healthy subjects, strain measurements derived from FT were lower (more negative) than the strain measurements determined with SENC. Furthermore, the range of strain values was greater when applying FT than when using SENC. The differences in strain measurements between SENC and STE-GLS and between Myo-FT and STE-FWLS were insignificant. These comparisons also showed the highest correlations. The calculated effect strengths were weak, with the highest being between Endo-FT and SENC and STE-GLS, showing that these approaches differed the most.

**Table 3:** Results of the Friedmann’s test to compare the different strain measurements in patients and healthy subjects, showing the adjusted significance level from the Dunn-Bonferoni post-hoc test (p) as well as the effect size (r) of the different comparisons

*Patients (n = 57)*	p	r
SENC vs. STE		
SENC vs. STE-FWLS	0.049	0.11
SENC vs. STE-GLS	1.000	/
FT vs. STE		
Endo-FT vs. STE -FWLS	< 0.001	0.18
Myo-FT vs. STE-FWLS	0.059	/
Endo-FT vs. STE-GLS	< 0.001	0.36
Myo-FT vs. STE-GLS	< 0.001	0.28
SENC vs. FT		
SENC vs. Endo-FT	< 0.001	0.29
SENC vs. Myo-FT	< 0.001	0.22
*Healthy subjects (n = 17)*		
SENC vs. FT		
SENC vs. Endo-FT	< 0.001	0.47
SENC vs. Myo-FT	0.006	0.26

*Abbreviations: GLS* Global longitudinal strain, *FWLS* Free wall longitudinal strain, *STE* Speckle tracking echocardiography, *SENC* Strain-encoding, *FT* Feature tracking, *Endo-FT* Subendocardial strain determined using FT, *Myo-FT* Midmyocardial strain determined using FT

### Reproducibility

The results of the reproducibility analyses can be seen in Table 4. Intra- and inter-observer reproducibility were excellent overall, particularly for STE and SENC (ICC ranging from 0.90–0.94 and CoV from 4 to 7%). The lowest CoV values were noted for SENC. FT showed only fair inter-observer reproducibility (ICC 0.62–0.67 and CoV from 29 to 30%) but better intra-observer reproducibility for midmyocardial strain (ICC 0.88, CoV 12%) as well.

**Table 4:** Results of the reproducibility analysis, reported in terms of intraclass correlation coefficient (ICC) and Coefficient of Variation (CoV)

		ICC (95%CI)	p	CoV (±SD)
Intra-Observer Reproducibility	STE-FWLS	0.94 (0.79–0.99)	< 0.001	0.07 (0.03)
	STE-GLS	0.93 (0.75–0.98)	< 0.001	0.08 (0.06)
	SENC	0.91 (0.71–0.98)	< 0.001	0.04 (0.03)
	Sub-FT	0.96 (0.85–0.99)	< 0.001	0.06 (0.05)
	Myo-FT	0.88 (0.50–0.97)	0.003	0.12 (0.10)
Inter-Observer Reproducibility	STE-FWLS	0.93 (0.75–0.98)	< 0.001	0.07 (0.03)
	STE-GLS	0.92 (0.72–0.98)	< 0.001	0.06 (0.06)
	SENC	0.90 (0.66–0.98)	< 0.001	0.04 (0.02)
	Endo-FT	0.62 (−0.22–0.91)	0.002	0.29 (0.11)
	Myo-FT	0.67 (−0.16–0.93)	< 0.001	0.30 (0.12)

*Abbreviations: GLS* Global longitudinal strain, *FWLS* Free wall longitudinal strain, *STE* Speckle tracking echocardiography, *SENC* Strain-encoding, *FT* Feature tracking, *Endo-FT* Subendocardial strain determined using FT, *Myo-FT* Midmyocardial strain determined using FT, *ICC* Intraclass correlation coefficient, *CI* Confidence interval, *CoV* Coefficient of variation, *SD* Standard deviation

## Discussion

The primary findings of our study are: 1) a modest but similar inter-modality agreement between each of the CMR techniques and STE. Furthermore, Myo-FT showed better agreement with both SENC and STE than Endo-FT. SENC showed better agreement with STE-GLS, whereas FT agreed more with STE-FWLS; 2) poor inter-technique agreement between SENC and FT with significant differences in the distribution of strain values in patients and healthy subjects, and 3) excellent intra- and inter-observer reproducibility, especially regarding STE and SENC, but only fair inter-observer reproducibility of FT-derived strain.

### Inter-modality agreement

Echocardiography is the most commonly used diagnostic tool for RV strain assessment due to its wide availability and low cost [5, 9]. However, the quality of echocardiographic images varies with the examiner’s experience and the constitution of the patient being imaged. CMR is less commonly used but also provides a variety of tools to measure RV strain. FT analysis can be performed on routinely acquired cine-bSSFP sequences, without prolonging the exam time. SENC, on the other hand, is a dedicated pulse-sequence, which can be acquired within a single heartbeat per view. Each of the techniques measure strain using different approaches. In this manuscript, we studied their relationship and found modest and very similar agreement between each of the CMR techniques and STE. The agreement between STE and FT (r = 0.50–0.62) that we observed was very similar to the agreement reported in patients with dilated cardiomyopathy (r = 0.64) [18] and with heart failure and reduced LVEF (r = 0.47) [15]. We found the differences in strain measurements between Myo-FT and STE-FWLS to be nonsignificant in the Dunn-Bonferoni post-hoc analysis, despite the moderate correlation (r = 0.62). Regarding the comparison between STE and SENC, we found a moderate agreement but no significant difference in the post-hoc analysis between strain measurements determined using SENC and STE-GLS, in comparison to FWLS. One previous study exists, which showed better agreement (r = 0.74) in a cohort of 30 patients with pulmonary hypertension [4], presumably because the increased RV wall thickness was associated with pulmonary hypertension.

There are various reasons that could explain our findings relative to what has been previously published. First, the RV is a thin walled structure; hence the spatial resolution of any imaging technique needs to be high enough to generate reliable strain measurements. With low spatial resolution, measurements are obtained from only a few pixels of data and there is a risk of including RV trabeculations, blood pool and pericardial fat in the strain analysis and not solely RV muscle. STE has a higher spatial resolution and temporal resolution than CMR, making it theoretically more reliable for imaging of the RV. However, this must be counterbalanced with the improved visualization of the RV wall offered by CMR compared to STE.

Secondly, due to the complex RV shape and its position being retrosternal and anterior to the LV [25], it may be difficult for 2-dimensional techniques such as STE, CMR-FT and SENC to fully capture RV strain. Furthermore, with echocardiography, the RV size and functional appearance could fluctuate depending on the imaging plane [25]. This could also be a potential advantage, as the imaging plane could potentially be adjusted for every patient to better grasp the complex shape of the RV. CMR image acquisition, however, is performed from standardized views [15], which could result in more consistent strain measurements.

Thirdly, during most of the echocardiographic and CMR exams, breath-holds are necessary. Depending on the patient’s breathing pattern during image acquisition, negative intrathoracic pressures increase RV preload, whereas any Valsalva maneuver would reduce preload [19]. Therefore, the breath-hold directly affects preload, which in turn affects RV strain. This factor does not only influence inter-modality agreement between CMR and STE, but might also contribute to the low inter-technique agreement between SENC and FT, since unlike FT, SENC can be acquired without the need for any breath-holds. Hence, “real-time” imaging techniques, such as SENC, might reflect a different aspect of biventricular interaction [19], which should be further investigated.

Lastly, the impact of different software packages for strain analysis should not be ignored [15]. Studies analyzing inter-vendor agreement have shown that different post-processing software packages may result in significantly different strain values [26–29]. In addition, different levels of physician experience [30] are also known to impact strain measurements.

### Layer-specific strain analysis

Our results show that Myo-FT correlated better with SENC and STE than Endo-FT. Hence, Myo-FT may be the more reliable approach based on our study cohort. Both software packages for SENC and STE automatically determine the midmyocardial strain between the epi- and endocardial contour, whereas only the FT software allows direct analysis of layer-specific strain. The contouring procedure and possibility of a layer-specific strain analysis varies with every software package, therefore it is important to further standardize post-processing, for example by publishing guidelines and consensus statements.

### Global vs. free-wall longitudinal strain

There is no clear consensus on whether RV strain analysis should include the septum or be determined as free-wall strain [5, 19]. For both SENC and FT, it was only possible to carry out strain analysis excluding the septum due to software settings. When comparing STE to SENC, the agreement in our study cohort was better for STE-GLS than STE-FWLS. When comparing FT with STE, FWLS showed marginally better agreement than GLS. This is not unexpected because FT-strain analyses also excluded the septum, but there also exists data showing better agreement between FT and STE-GLS than STE-FWLS [20]. Particularly in diseases with reduced LV function, global RV strain values including the septum are likely to be biased by LV strain, however this does not have to be a disadvantage. In fact, in patients with heart failure and reduced EF, contradicting results were recently published on which of the parameters might have the better predictive power [15, 31]. Future research should thoroughly assess the impact of including the septum on RV strain in different diseases, but to date, both approaches seem to be equally meaningful to determine RV function.

### Inter-technique agreement and reproducibility

In our study, although STE showed modest agreement with both FT and SENC, the CMR techniques agreed poorly with one another. Moreover, a bias in strain measurements was noted with more negative strain values determined by FT than by SENC in patients and in healthy subjects. These results are similar to the distribution of strain values reported by others [32, 33]. We also found suboptimal reproducibility between different observers for FT in comparison to SENC. Several factors might influence this relationship. Firstly, as mentioned above, the patient’s breathing during image acquisition directly affects RV preload. During SENC-acquisition, breath holds are not necessary and image acquisition time is one heartbeat per view [34, 35], whereas for cine-bSSFP- image acquisition breath holds are usually necessary and image acquisition cannot be performed within a single heartbeat [35]. Secondly, FT is susceptible to artefacts caused by through-plane motion [32]. This might also explain the narrow range of strain values determined with SENC compared to FT in both patients and volunteers. Moreover, SENC uses out-of-plane phase encoding gradients orthogonal to the image plane [17, 36]. Hence, LS is determined from the strain-encoded short-axis images, whereas the FT software employed for this analysis determines LS from the 4-Ch long-axis view only. Furthermore, SENC RV strain measurements madein the short-axis view exclude the apex, whereas FT measurements include the apex. Also, the accuracy of FT heavily relies on correctly tracking the endocardial border, which is particularly difficult in case of the thin RV walls. In contrast, accurate tracking is not needed for SENC, which relies on frequency shifts seen in K-space. Finally, differences in the post-processing software also need to be considered. Both software packages use unique algorithms to calculate global strain, which are proprietary. Moreover, the software packages do not provide information on layer-specific strain analysis. Whereas currently SENC analysis can only be performed using the endo- and epicardial layer (midmyocardial strain), the FT software also allows for endocardial strain calculation separately. This is the first study to compare inter-technique agreement between SENC and FT for the RV; hence more research is needed to evaluate the impact of all the above factors on the relationship between the two techniques. Also, potential use of different technique-specific normal values should be considered to increase comparability when used clinically.

#### Limitations

The study is retrospective with a small cohort of patients. Additionally, patients with a wide range of cardiac diseases were included, therefore we cannot relate our results to a specific patient group. We only determined LS, as it has been shown previously that LS reflects the contractile function of the RV better than circumferential strain [37].

## Conclusions

There are modest correlations between both CMR-derived FT and SENC and STE measurements of RV-LS, whereas FT and SENC correlated weakly with one another. Although our results show that most of the studied approaches are highly reproducible, the measurements are not interchangeable. Additional effort is needed to facilitate comparisons between RV strain measurements made using different imaging modalities and techniques, and post-processing methods should be more uniform with regards to their analytical approaches to strain analysis and their strain calculation algorithms.

## Data Availability

Data supporting the results reported in the manuscript can be found in a computer workstation in the Cardiac Imaging Laboratory at the University of Chicago.

## Abbreviations

4Ch: Four-chamber
BSA: Body surface area
bSSFP: Balanced steady state free precession
CMR: Cardiovascular magnetic resonance
ED: End-diastole
EF: Ejection fraction
Endo-FT: Subendocardial feature tracking strain
ES: End-systole
FT: Feature tracking
FWLS: Free wall longitudinal strain
GLS: Global longitudinal strain
HFpEF: Heart failure with preserved ejection fraction
HFrEF: Heart failure with REDUCED ejection fraction
LoA: Limits of agreement
LS: Longitudinal strain
LV: Left ventricle/left ventricular
LVEF: Left ventricular ejection fraction
Myo-FT: Mid-myocardial strain using feature tracking
RV: Right ventricle, right ventricular
RVEF: Right ventricular ejection fraction
SENC: Strain encoding
STE: Speckle tracking echocardiography
